# Identification of developmentally-specific kinotypes and mechanisms of Varroa mite resistance through whole-organism, kinome analysis of honeybee

**DOI:** 10.3389/fgene.2014.00139

**Published:** 2014-05-21

**Authors:** Albert J. Robertson, Brett Trost, Erin Scruten, Thomas Robertson, Mohammad Mostajeran, Wayne Connor, Anthony Kusalik, Philip Griebel, Scott Napper

**Affiliations:** ^1^Meadow Ridge Enterprises Ltd.Saskatoon, SK, Canada; ^2^Department of Computer Science, University of SaskatchewanSaskatoon, SK, Canada; ^3^Vaccine and Infectious Disease Organization, University of SaskatchewanSaskatoon, SK, Canada; ^4^School of Public Health, University of SaskatchewanSaskatoon, SK, Canada; ^5^Department of Biochemistry, University of SaskatchewanSaskatoon, SK, Canada

**Keywords:** peptide arrays, kinome, kinotype, *Varroa destructor*, honeybee, *Apis mellifera*

## Abstract

Recent investigations associate *Varroa destructor* (Mesostigmata: Varroidae) parasitism and its associated pathogens and agricultural pesticides with negative effects on colony health, resulting in sporadic global declines in domestic honeybee (*Apis mellifera*) populations. These events have motivated efforts to develop research tools that can offer insight into the causes of declining bee health as well as identify biomarkers to guide breeding programs. Here we report the development of a bee-specific peptide array for characterizing global cellular kinase activity in whole bee extracts. The arrays reveal distinct, developmentally-specific signaling profiles between bees with differential susceptibility to infestation by Varroa mites. Gene ontology analysis of the differentially phosphorylated peptides indicates that the differential susceptibility to Varroa mite infestation does not reflect compromised immunity; rather, there is evidence for mite-mediated immune suppression within the susceptible phenotype that may reduce the ability of these bees to counter secondary viral infections. This hypothesis is supported by the demonstration of more diverse viral infections in mite-infested, susceptible adult bees. The bee-specific peptide arrays are an effective tool for understanding the molecular basis of this complex phenotype as well as for the discovery and utilization of phosphorylation biomarkers for breeding programs.

## 1. Introduction

In recent years, there has been an alarming worldwide decline in populations of honeybees (*Apis mellifera*) (Dietemann et al., 2013). This is of considerable concern, as approximately one-third of the human food supply depends on pollination by the honeybee (Greenleaf and Kremen, 2006; Cox-Foster et al., 2007; Vanengelsdorp et al., 2009). A number of possible causes have been suggested, including Varroa mite parasitism and associated pathogens (Martin et al., 2012; Nazzi et al., 2012), increased use of pesticides, lack of genetic diversity, and other factors (Vanengelsdorp et al., 2009; Mullin et al., 2010).

The ectoparasitic mite *Varroa destructor*, and RNA viruses that are associated with it, are a significant challenge to the honeybee. Deformed wing virus (DWV) (Martin et al., 2012, 2013), Israeli acute paralysis virus (IAPV), acute bee paralysis virus (ABPV), and Kashmir bee virus (KBV) are the major viruses vectored by Varroa (Di Prisco et al., 2011). Varroa mites continue to spread throughout the world and contribute to the decline of domesticated honeybee populations (Martin et al., 2012; Nazzi et al., 2012). Their natural host, the Asian honeybee (*Apis ceranae*), has developed protective mechanisms based on behavioral characteristics, such as grooming and hygienic traits, as well as differences in brood development time, rather than differences in immunity (Sammataro et al., 2000; Rosenkranz et al., 2010). The western honeybee, initially exposed to Varroa mite parasitism in the mid-1960s (Sammataro et al., 2000), has yet to develop adequate resistance mechanisms. Many synthetic miticides have been deployed to combat Varroa infestations, but the mites quickly develop resistance; further, the miticides have detrimental effects on honeybee health, and can also leave dangerous residues in the wax (Lodesani and Costa, 2005).

A more attractive approach is to breed honeybees capable of resisting or controlling Varroa mite infestation. However, breeding for Varroa resistance is complicated by a lack of understanding of honeybee susceptibility to mite parasitism, a dearth of biomarkers to identify potentially resistant progeny, and the instability of resistant phenotypes. A number of groups have used natural selection to identify colony phenotypes with Varroa resistance (Le Conte et al., 2007; Seeley, 2007). The most well-characterized genetic stocks able to suppress Varroa population growth are the Varroa sensitive hygiene (VSH) lines (Harbo and Harris, 2009; Tsuruda et al., 2012). In this work, the Saskatraz natural selection project (http://www.saskatraz.com) selected and characterized susceptible and resistant honeybee colony phenotypes for molecular analyses. This project focuses on recurrent natural selection of survivor colonies for honey production, wintering ability, resistance to Varroa, and overall colony health, in the absence of synthetic miticides.

There is a general consensus that understanding the cellular mechanisms of these disease-resistance phenotypes requires a global perspective on bee biology. To this end, a number of recent studies have examined the differential expression of genes (Le Conte et al., 2011) and proteins (Parker et al., 2012) in honeybees that suppress Varroa population growth. These efforts have neither provided clear insight into the cellular mechanisms of Varroa mite susceptibility nor identified reliable biomarkers. This reflects the challenges associated with deciphering complex biology, in particular within the context of a mixed genetic population.

Similar challenges have been overcome in other livestock species through the development and application of species-specific peptide arrays for analysis of global cellular kinase (kinome) activity (Arsenault et al., 2012, 2013b; Trost et al., 2013a). Kinase-mediated protein phosphorylation is critical for the regulation of cellular responses and phenotypes. Analysis of global kinome activity has provided a powerful tool to understand complex biology as well as to identify therapeutic targets and biomarkers (Eglen and Reisine, 2011). In particular, the ability to use short peptides as surrogate substrates for kinases makes it possible to monitor the kinome using high-throughput peptide arrays (Arsenault et al., 2011). While detailed descriptions of the phosphoproteome are available for only a limited number of species, it is possible to predict the sequence contexts of phosphorylation events based on genomic information, creating the opportunity to develop species-specific kinome microarrays for species whose phosphoproteomes have not been extensively characterized (Jalal et al., 2009; Trost et al., 2013a). Kinome analysis has been demonstrated to have considerable utility in understanding cellular mechanisms of host-pathogen interaction (Kindrachuk et al., 2011; Arsenault et al., 2012, 2013a; Määttänen et al., 2013; Mulongo et al., 2014) as well as identifying phosphorylation biomarkers that predict or reflect phenotypic traits (Arsenault et al., 2013b). Recently, the existence of temporally-stable species and individual-specific phosphorylation profiles, or kinotypes, was reported (Trost et al., 2013c). These stable patterns within individuals likely reflect genetic, epigenetic, environmental and developmental influences and may provide mechanistic and predictive insight into complex, multi-factorial phenotypes. Similarly, while kinome analysis is traditionally performed on samples of low biological complexity, such as cultured cells or purified cell populations, recent applications have extended this analysis to more complex samples, including intestinal tissue (Määttänen et al., 2013) and muscle biopsies (Arsenault et al., 2013b).

Here we report the development of a bee-specific kinome array and its application to characterize honeybees with a quantified, differential susceptibility to Varroa mite infestation. Bees of the susceptible and resistant phenotypes possess distinct kinome profiles at a number of developmental stages ranging from pupae to adult, highlighting the potential to use these differences as markers for breeding programs. Kinome analysis also offers insight into the mechanisms underlying disease susceptibility. Specifically, the kinome data indicate that the susceptibility to Varroa mite infestation does not reflect compromised immunity. There is, however, evidence for mite-mediated immune suppression within the susceptible phenotype, which may reduce the ability of these bees to counter secondary infections. Consistent with this hypothesis, an increased diversity of viral infections is observed in Varroa-infested susceptible bees. Overall, the bee-specific peptide arrays offer an effective tool for understanding the molecular basis of complex phenotypes and for analyzing specific biological responses, and may facilitate the identification of phosphorylation biomarkers for breeding programs.

## 2. Materials and methods

### 2.1. Colony phenotype selection

A detailed description of the honeybee breeding and selection program that was used to construct and identify the Varroa mite susceptible and resistant phenotypes can be accessed at http://www.saskatraz.com. Briefly, Meadow Ridge Enterprises Ltd. established a closed-population mating program in 1992, selecting from approximately 1200 colonies annually for honey production, wintering ability and chalk brood resistance. Tracheal mites were first observed in the colonies in the late 1990s, and Varroa mites were detected shortly thereafter. The selected population showed no resistance to either mite. To introduce mite resistance, Russian stock was imported as embryos from the USDA between 2001 and 2005 (Rinderer et al., 2001). Russian virgins from three different selections were close-population mated to selected colonies at the Meadow Ridge apiary. The F1 hybrids from these initial crosses were established at three different isolated apiaries, and used to backcross Russian virgins from subsequent shipments to regenerate Russian stock, and for re-selection under Canadian conditions. These apiaries served as a source of colonies for the natural selection apiary, and for drones in crosses used to increase Varroa resistance. In 2004, a natural selection apiary was established at an isolated area in Saskatchewan, called Saskatraz, using colonies from Meadow Ridge and collaborating Saskatchewan beekeepers. This apiary was established to further select for productive colonies with mite resistance and good wintering ability, without synthetic miticide treatment. Tracheal mites were introduced in the fall of 2004 by adding 200 worker bees with 60% tracheal mite infestations. Varroa mites were present in the original selections.

A colony phenotype called Saskatraz 88 (S88) was constructed by backcrossing a daughter from a Russian hybrid line selected at Saskatraz in 2006 to drones at an isolated Russian apiary (RP30) previously established at Meadow Ridge to increase Varroa tolerance. The resulting colony superseded and a daughter was mated at the RP30 apiary again, resulting in two back crosses at the RP30 apiary. Extensive screening of Varroa present on adult bee populations in both breeding populations and commercial colonies identified G4, a susceptible colony phenotype established in the summer of 2009. G4 bees showing high Varroa mite infestations during spring evaluations were selected and moved to an isolated apiary used as a Varroa nursery for experimental purposes. Susceptible colonies were not treated and left to die, serving to remove susceptible colonies from the breeding population. G4 and S88 were located in different apiaries during the course of the experiment. No queen events (swarming, supersedure) were noted in either S88 or G4 colonies during their lifespans. The S88 queen was last observed in the fall of 2010 in the Saskatraz natural selection apiary and failed in the spring of 2011.

Varroa infestations on adult bees (phoretic phase) were evaluated by washing 200–300 bees in 100% methanol. Analyses of Varroa in sealed brood (percent brood infestation and number of Varroa per cell) and natural Varroa drop onto sticky boards was also monitored. For molecular analyses, several hundred adult worker bees were collected from the brood nest and white-eyed, pink-eyed and dark-eyed pupae were collected from sealed brood of both S88 and G4 colonies in September 2010. Pupae and adult bees, either infested or not infested with Varroa mites, were collected. The samples were frozen in liquid nitrogen and stored at −80°C.

### 2.2. Design of a honeybee-specific peptide array

To the authors' knowledge, no phosphorylation sites have been experimentally characterized in honeybee. As such, the following procedure was performed in order to identify putative honeybee phosphorylation sites. Experimentally-determined phosphorylation sites from other organisms were downloaded from the PhosphoSitePlus (Hornbeck et al., 2004, 2012) and Phospho.ELM (Diella et al., 2004, 2008; Dinkel et al., 2011) databases, and were combined into a single file. These included sites from organisms such as human, rat, mouse, cow, and *Drosophila melanogaster* (the closest honeybee relative for which phosphorylation sites are known). Phosphorylation sites were represented as 15-mer peptides, with the phosphorylated residue in the center and seven residues on either side. The honeybee proteome was constructed as follows. First, all of the honeybee proteins from UniProt (671 proteins) and GenBank (12,050 proteins) were downloaded. Second, the honeybee genome (Honeybee Genome Sequencing Consortium, 2006) was downloaded in the form of 16,501 contigs, and genes (along with their translations) were predicted using the program GeneMark.hmm (Lukashin and Borodovsky, 1998), giving 27,730 predicted proteins. Proteins from these three sources were then combined to create a final honeybee proteome consisting of 40,451 proteins. Using the DAPPLE program (Trost et al., 2013a), the 15-mer peptides from PhosphoSitePlus and Phospho.ELM were searched using BLAST against the honeybee proteome to find homologous sites. DAPPLE produced a table designed to facilitate the process of selecting honeybee peptides for inclusion on the array. Each row of the output table corresponded to a phosphorylation site from PhosphoSitePlus or Phospho.ELM. In addition to the sequence of the best hit in the honeybee proteome, the table contained the number of sequence differences between the query peptide and the honeybee peptide, with honeybee peptides having few sequence differences being preferred. The table also included the position (e.g., Y128) of the phosphoacceptor residue for both the query peptide and the hit peptide, with honeybee peptides where the position was similar for both query and hit being preferentially selected. In addition, peptide sequences contained within proteins from UniProt or GenBank were preferred over those from proteins predicted by GeneMark.hmm. Using the above criteria, this list was manually curated to select appropriate honeybee phosphorylation sites for inclusion on the array. Peptides were selected that represent phosphorylation events associated with a broad spectrum of signaling pathways, but with specific emphasis on proteins and processes associated with innate immunity. A total of 299 peptides were ultimately selected. Each of these peptides was spotted in triplicate within each block. Further, each block was printed in triplicate, providing nine technical replicates for each peptide. Peptide synthesis, array spotting and quality control were performed as a commercial service (JPT Peptide Technologies, Berlin, Germany).

### 2.3. Kinome analysis

Application of the peptide arrays was based upon a previously reported protocol with modifications (Määttänen et al., 2013). Briefly, individual frozen whole bees were placed in a sealed plastic bag in the presence of 300 μl of lysis buffer. The bees were struck repeatedly with a rubber mallet and the suspension was centrifuged at 10,000 × g for 10 min. Supernatants were used for kinome analysis.

### 2.4. Data analysis

The dataset for each array contained the signal intensities associated with the nine technical replicates for each of the 299 peptides for the whole body extracts of honeybee pupae or adults either uninfested or infested with Varroa mites. Those treatments were labeled “G4−” (susceptible and uninfested), “G4+” (susceptible and infested), “S88−” (resistant and uninfested), and “S88+” (resistant and infested). Kinome data were processed through PIIKA 2, a pipeline for processing kinome array data (Li et al., 2012; Trost et al., 2013b), with the following study specifics.

#### 2.4.1. Consistency of technical replicates

For each peptide within a given array, a chi-square test was performed to determine whether the degree of variability among the technical replicates for that peptide was greater than would be expected by chance. Any peptide that had a *P*-value according to the chi-square test of less than 0.01 was considered to be inconsistently phosphorylated among the technical replicates.

#### 2.4.2. Treatment-treatment variability analysis and pathway analysis

For each peptide, a paired *t*-test was used to compare its normalized signal intensity values under a treatment condition with those under a control condition. Three tests were performed for each peptide: G4+ versus G4−, S88+ versus S88−, and G4− versus S88−. Peptides with significant (*P*-value < 0.10) changes in phosphorylation were identified. This level of significance was chosen to retain as much data as possible in order to facilitate subsequent pathway analysis (Li et al., 2012). Pathway and gene ontology (GO) analysis was performed as described previously (Kindrachuk et al., 2011; Määttänen et al., 2013) using InnateDB (Lynn et al., 2008).

#### 2.4.3. Cluster analysis

The pre-processed data were subjected to hierarchical clustering and principal component analysis (PCA) to cluster peptide response profiles across arrays. Only peptides that were consistently phosphorylated among the technical replicates for all arrays were included in the clustering analysis. For each consistently-phosphorylated peptide on a given array, the average was taken over the nine replicates before performing clustering. For hierarchical clustering, the distance metric used was (1−Pearson correlation), while the linkage method used was that of McQuitty (1966). Subsets of peptides that could discriminate between resistant and susceptible bees were identified as described previously (Trost et al., 2013b).

### 2.5. Virus detection

Bees were stored at −80°C until RNA was extracted. Individual pupa were placed in small plastic bags, pulverized on dry ice, and solubilized in 700 μl Trizol (Invitrogen Canada, Burlington, ON). RNA was purified using RNeasy Mini-columns (Qiagen Canada Inc., Mississauga, ON) and RNA concentration quantified with an Agilent 2100 Bioanalyzer using RNA 6000 Nano kits (Agilent Technologies Canada Inc., Mississauga, ON). RNA pellets were re-suspended in DEPC water and converted to cDNA using qScript cDNA Supermix (Quanta Biosciences, Gaithersburg, MD). qRT-PCR was performed using PerfeCta SYBR Green Supermix for IQ (Quanta Biosciences) on a BioRad IQ5 thermocycler. Deformed wing virus was detected using primers CAGTAGCTTGGGCGATTGTT (forward) and AGCTTCTGGAACGGCAGATA (reverse) (Cox-Foster et al., 2007). Israeli acute paralysis virus was detected using primers GCGGAGAATATAAGGCTCAG (forward) and CTTGCAAGATAAGAAAGGGGG (reverse) (Di Prisco et al., 2011). Kashmir bee virus was detected using primers GATGAACGTCGACCTATTGA (forward) and TGTGGGTTGGCTATGAGTCA (reverse) (Cox-Foster et al., 2007). The presence of a single PCR product of the expected size was confirmed in 2% agarose gels (Invitrogen). Detection of DWV, IAPV, and KBV was performed using an end-point PCR protocol with Phusion polymerase (New England Biolabs, Whitby, ON) with amplification at 98°C for 30 s, then 30 cycles of: 98°C for 10 s, 60°C for 15 s, and 72°C for 20 s followed by 20 s at 72°C. Amplified products were visualized with ethidium bromide staining of 2% agarose gels. The real time cycling protocol for quantification of DWV was 95°C for 2 min, then 40 cycles of 95°C for 15 s, 60°C for 30 s, and 72°C for 30 s, followed by a melt curve to confirm amplification of a single product.

## 3. Results

### 3.1. Characterization of varroa mite susceptible and resistant bee phenotypes

**Figure 1:**
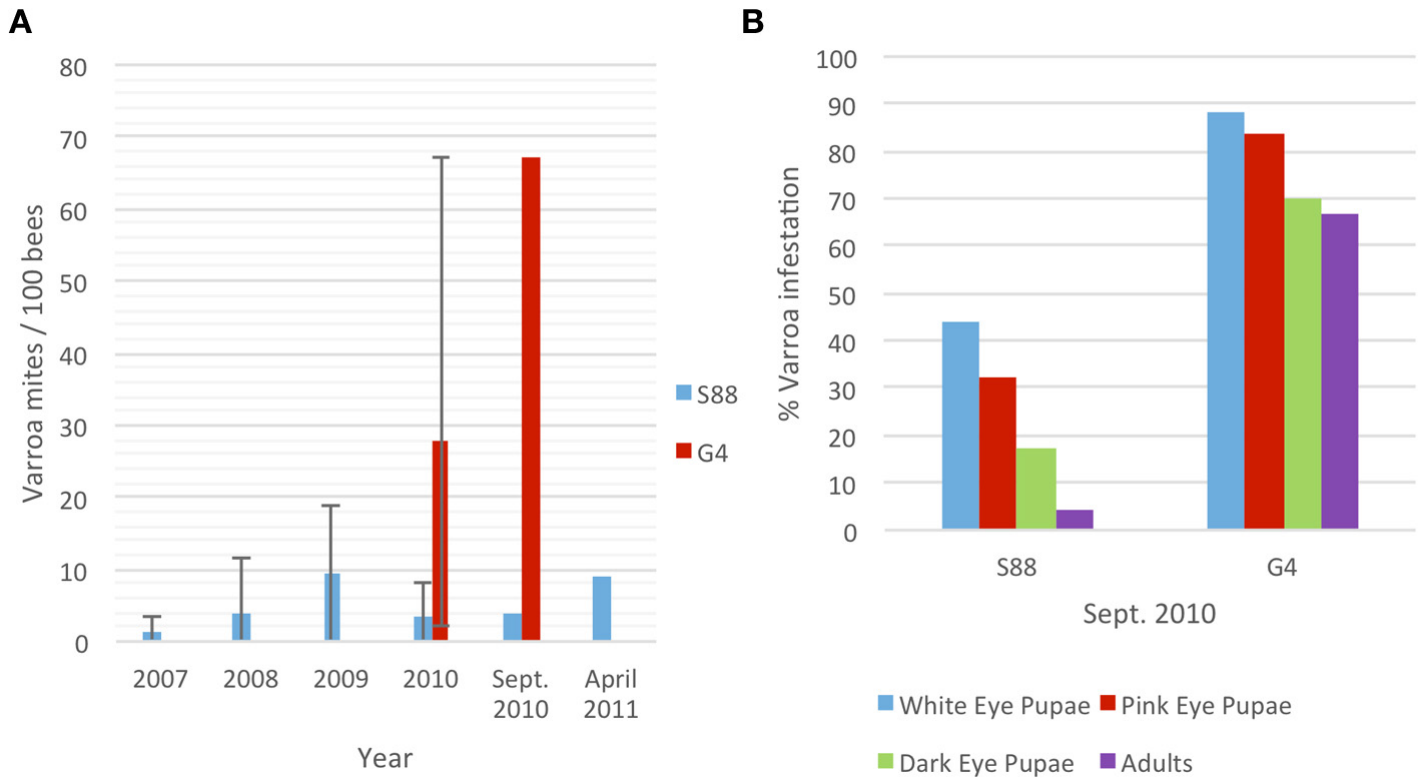
**Quantification of Varroa mite infestation of G4 and S88 bees. (A)** Average phoretic Varroa infestations per 100 bees in S88 and G4 colonies. Bars show the range of yearly phoretic Varroa infestations in S88 (2007–2010) and G4 (2010). **(B)** Percent Varroa infestation in sealed brood at different stages of development. Over 500 sealed brood cells were analyzed for each colony and scored for presence of Varroa.

Varroa mite infestation was quantified yearly between 2007 and 2011 for the resistant (S88) colony and in 2010 for the susceptible (G4) colony (Figure 1A). In 2009, the average Varroa infestation rates for S88 remained below 10 per 100 bees (PHB) but ranged as high as 19 PHB. In 2010, eight samples were analyzed between May and October showing an average infestation of three to five PHB in the S88 colony. Adult bee samples with and without Varroa were sampled in September for kinome analyses, when phoretic mite levels were four PHB (Figure 1A). S88 died in April 2011 with a Varroa mite population of nine PHB after a colony lifespan of 58 months. This colony resisted Varroa mite population growth throughout its lifetime, although significant levels of Varroa mites persisted in the colony from establishment. High levels of phoretic Varroa were detected in May 2010 in G4 and reached as high as 67 PHB. Varroa mite population growth was very rapid in this colony (Figure 1A). Adult bees with and without Varroa were sampled for kinome analyses when phoretic Varroa populations were highest (September 2010). G4 died in October with a lifespan of 17 months.

These resistant and susceptible colonies were further defined by evaluating Varroa infestation in the sealed brood at the same time as adult bee samples were collected for molecular analyses. Honeybee colonies during September in Western Canada decrease brood rearing and the adult population begins to decline. Varroa increase migration into the brood, and brood Varroa levels can quickly increase. Scoring sealed G4 brood cells (*n* = 500) revealed that 88%, 84%, and 70% of the white-eyed, pink-eyed and dark-eyed pupae, respectively, were Varroa-infested (Figure 1B). The phoretic mite levels on adult G4 bees (67 PHB) was similar to the infestation rate for dark-eyed pupae. In contrast, S88 brood infestation levels were much lower, with dark-eyed pupae infestation levels dropping to 17% from 44% and adult phoretic levels to four PHB (Figure 1B). These results imply that S88 resists Varroa population growth by removing Varroa from the brood. In addition, fewer Varroa per cell were detected in dark-eyed pupae and pre-emergent pupae in S88 than G4 at July 2010 sampling dates. G4 showed 2.7 ± 2.0 Varroa per cell (± standard error of the mean, *n* = 70), and S88 showed 1.5 ± 1.0 Varroa per cell (*n* = 9).

### 3.2. Development of a bee-specific peptide array

The bee-specific peptide array was developed using the DAPPLE program (Trost et al., 2013a) as described in section 2. DAPPLE predicted nearly 10,000 phosphorylation events within the honeybee proteome. Of the predicted phosphorylation events, approximately 0.6% were exactly conserved over a peptide of 15 amino acids (seven residues flanking each side of the phosphoacceptor site) (Supplementary Table 1). The low degree of conservation highlights the importance of developing species-specific arrays as opposed to simply translating commercially available arrays across species.

From this panel, 299 unique phosphorylation events were selected using the criteria described in section 2. Peptides were selected to represent phosphorylation events associated with a broad spectrum of signaling pathways (to facilitate novel discovery) but with emphasis on pathways and processes associated with insect innate immunity. A GenePix Array List (GAL) file containing the exact layout and content of the array used in this study is provided (Supplementary File 1).

**Figure 2:**
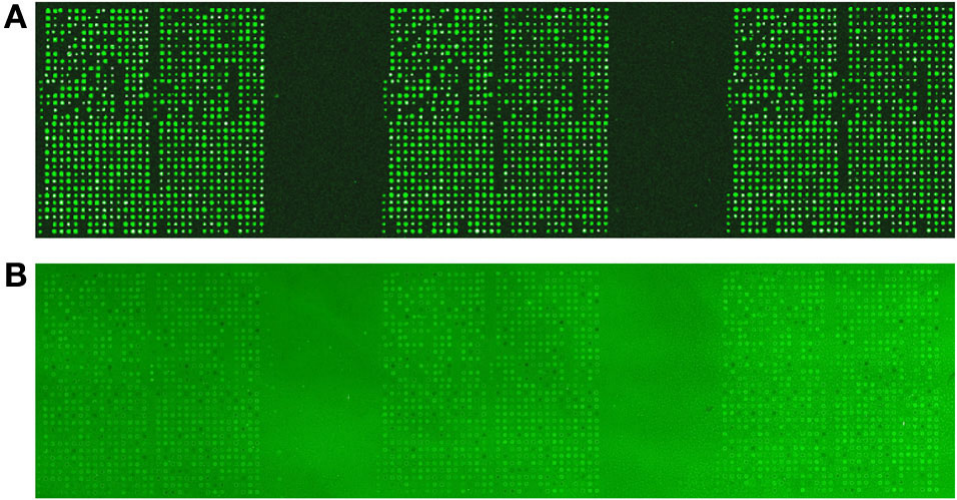
**Printing and validation of the bee-specific peptide array. (A)** The arrays were printed by a commercial provider (JPT Peptide Technologies, Berlin, Germany). For each array, each spot was printed in triplicate within each block. Each block was then printed in triplicate, for a total of nine technical replicates of each peptide. This image, taken as a quality control step in array production, illustrates the consistency and reproducibility of peptide spotting. **(B)** An image of a data scan of a representative array used for analysis of a whole-bee sample. A clear and consistent pattern of peptide phosphorylation is apparent across the three printed blocks.

An image highlighting the format of the arrays as well as the consistency and reproducibility of peptide spotting is presented (Figure 2A). An image of a data scan of a representative array used for analysis of a whole-bee lysate is also provided (Figure 2B). All of the arrays used in this study were of comparable quality with respect to the clarity and consistency of peptide phosphorylation.

### 3.3. Kinome profiling of bee phenotype at different developmental stages

**Figure 3:**
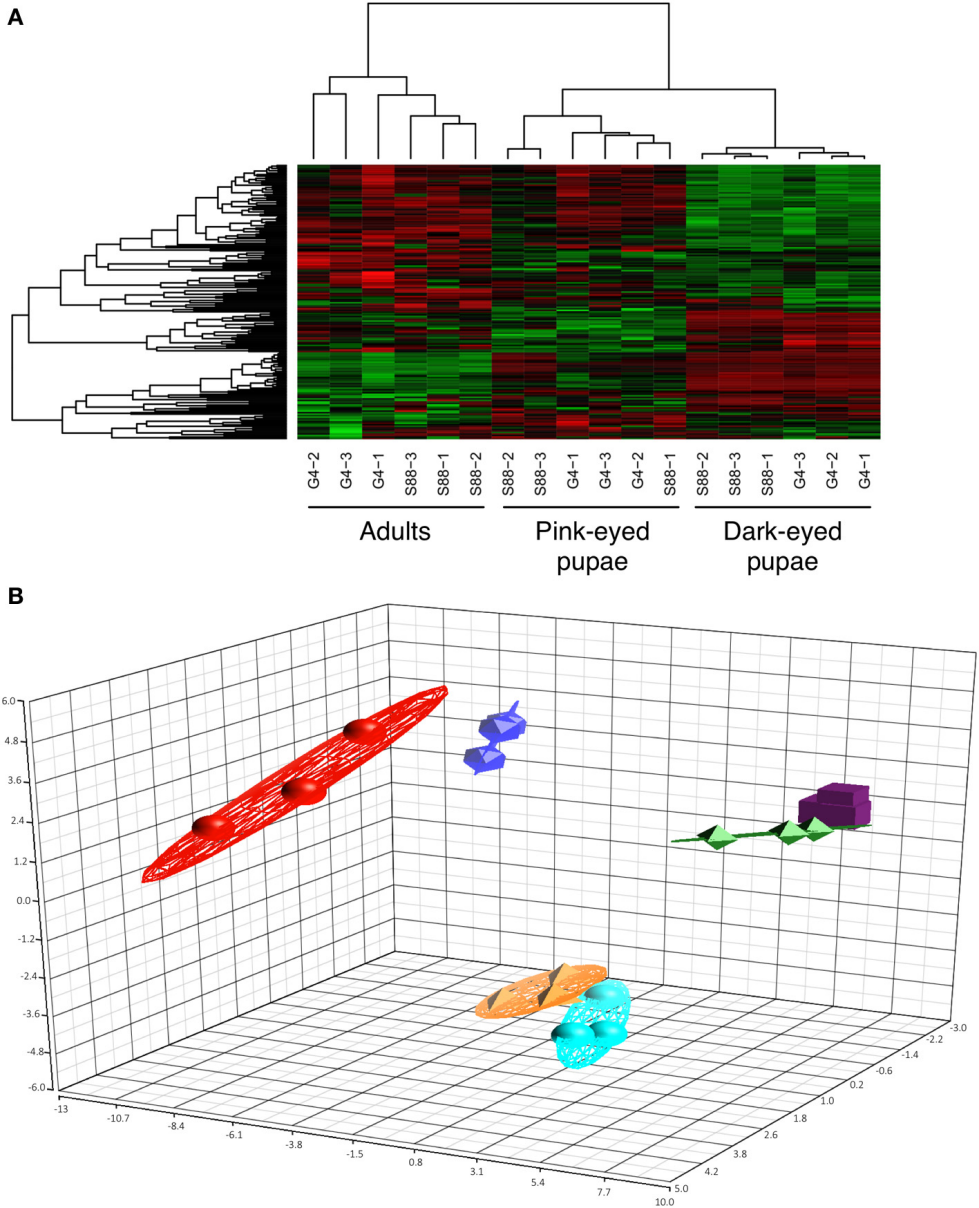
**Clustering of the kinome profiles of bees of different phenotypes at different developmental stages. (A)** Hierarchical clustering of kinome datasets. (1−Pearson correlation) was used as the distance metric, while McQuitty linkage was used as the linkage method. Each column represents the kinome activity of individual bees (*n* = 3/treatment). The kinome profiles of the bees segregated first by developmental stage and then largely by colony phenotype (S88: resistant; G4: susceptible). Colors indicate the average (over nine intra-array replicates) normalized phosphorylation intensity of each target, with red indicating greater amounts of phosphorylation and green indicating lesser amounts of phosphorylation. **(B)** Principal component analysis. The first three principal components are shown. The points are as follows: red, adult G4; dark blue, adult S88; green, dark-eyed G4; purple, dark-eyed S88; orange, pink-eyed G4; light blue, pink-eyed S88. The proportions of variance explained by the first, second, and third principal components were 29.1%, 15.3%, and 7.5%, respectively.

Uninfested bees (*n* = 3) of each phenotype (G4 and S88) were considered at each of three developmental stages (pink-eyed pupae, dark-eyed pupae and adult). In each case, kinome analysis was performed with lysate extracted from the whole organism. Morphologically, there was a clear distinction between each developmental stage. There was, however, no obvious difference in bee morphology when comparing between G4 and S88 within each development stage. The relationships among the 18 kinome datasets were evaluated through hierarchical clustering (Figure 3A) and three-dimensional PCA (Figure 3B). There was a clear indication of distinct developmentally-specific kinome profiles. Further, within each developmental stage, there was strong evidence of distinct kinome profiles for the G4 and S88 bees, indicating that Varroa mite susceptibility or resistance is reflected at the level of signal transduction.

### 3.4. Phosphomarkers of varroa mite susceptibility in dark-eyed pupae

The ability of the arrays to detect distinct kinome profiles (kinotypes) corresponding to each phenotype suggests that the arrays may represent a valuable tool for identification of kinase activity biomarkers that are associated with resistance or the response to Varroa mite infestation. Specifically, the bee-specific peptide array, representing 299 phosphorylation events, was able to discriminate between each developmental stage, and between the two phenotypes within each developmental stage (Figure 3).

To determine whether smaller sets of peptides could also discriminate between the phenotypes, the peptide subset analysis described by Trost et al. (2013b) was performed on the bees at the dark-eyed pupae stage. This procedure was used to identify subsets of peptides having the property that, when samples were clustered using these peptides, bees of the same phenotype clustered together as closely as possible. This was done for peptide subsets of size 3–200. For subsets of selected cardinalities (5, 10, 25, 50, 100, 150, and 200), the random tree analysis described by Trost et al. (2013b) was performed to determine whether that set of peptides discriminated between the susceptible and resistant phenotypes better than would be expected by chance. It was discovered that subsets of as few as five peptides could discriminate the resistant and susceptible bee phenotypes with a high degree of confidence (*P*-value < 0.001) (Table 1). Given this, it may be possible to create a smaller, more targeted array that could provide unique kinomic profiles for each phenotype. Such a peptide subset could serve as a minimal array of practical value for screening bees within breeding programs as well as for assurance of phenotype in the sales and marketing of commercial bees.

**Table 1:** **Ability of subsets of peptides to discriminate susceptible and resistant bees at the dark-eyed pupae stage**.

**Number of peptides**	***P*-value**
200	0.0006
150	0.0002
100	0.0007
50	0.0001
25	0.0002
10	0.0004

*Subsets of peptides were determined that best differentiated susceptible and resistant dark-eyed pupae. For selected subsets, a statistical test (Trost et al., 2013b) was used to determine whether those peptides could discriminate between the two phenotypes better than would be expected by chance. The first column of the table contains the size of the peptide subset, while the second column contains the P-value associated with this statistical test*.

### 3.5. Kinomic responses of susceptible and resistant dark-eyed pupae to varroa mite challenge

**Figure 4:**
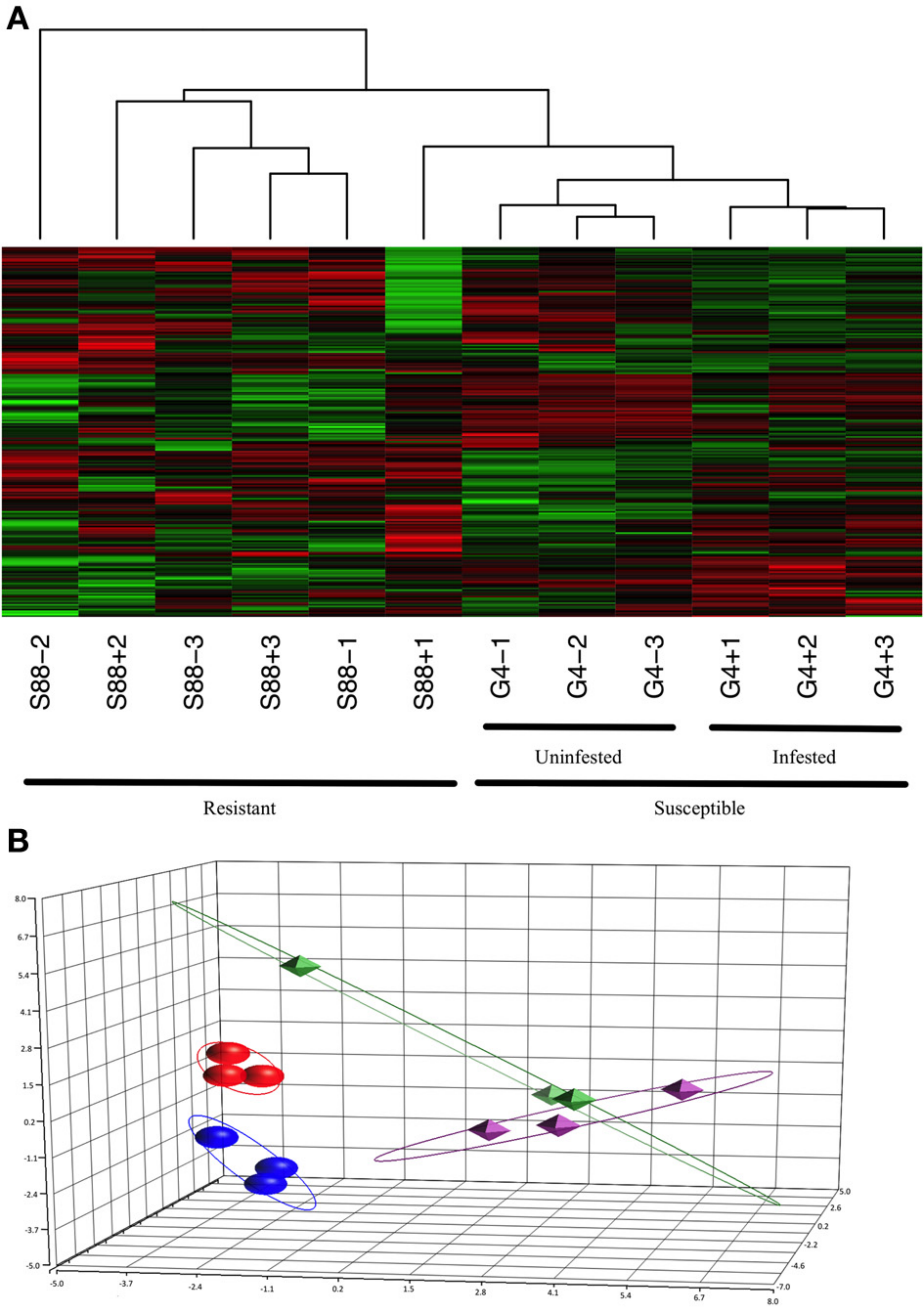
**Clustering of the kinome profiles of dark-eyed pupae of different phenotypes and infestation statuses. (A)** Hierarchical clustering of kinome datasets. (1−Pearson correlation) was used as the distance metric, while McQuitty linkage was used as the linkage method. Each column represents the kinome activity of individual pupae (*n* = 3/treatment). For the most part, cluster analysis first segregated kinome profiles by colony phenotype (S88: resistant; G4: susceptible), and then segregated G4 pupae by presence or absence of Varroa infestation. **(B)** Principal component analysis. The first three principal components are shown. Separation of the samples on the basis of phenotype is clearly observed, with further distinction within the susceptible, but not resistant, samples on the basis of infestation status. The points are as follows: red, G4+; dark blue, G4−; green, S88+; purple, S88−. The proportions of variance explained by the first, second, and third principal components were 22.5%, 14.8%, and 11.2%, respectively.

Kinome profiles were determined for individual dark-eyed pupae (*n* = 3) of both the G4 and S88 colony phenotypes in the presence and absence of Varroa mite infestation. Hierarchical clustering analysis of the kinome data demonstrated distinct clustering on the basis of Varroa mite susceptibility, indicating distinct patterns of phosphorylation-mediated signal transduction within the two phenotypes (Figure 4A). This was confirmed with PCA, in which distinct clustering of samples corresponding to the phenotypes was also observed (Figure 4B). For both hierarchical clustering and PCA, there was further sub-clustering based on the infestation status of the samples within the susceptible phenotype. This sub-clustering was not observed within the resistant samples, except for one S88 infested pupae which showed some overlap with the susceptible G4 phenotype. These observations imply Varroa parasitism induced a more pronounced change in intracellular physiology within Varroa susceptible bees compared to resistant bees.

### 3.6. Cellular mechanisms of varroa mite susceptibility

The kinome data were interrogated to define the biological differences between bee phenotypes at the dark-eyed pupae stage of development. Many peptides were differentially phosphorylated between phenotypes or treatments. For instance, in the uninfested samples of each phenotype, there were 153 peptides (over half of the peptides on the array) for which there were significant (*P*-value < 0.1) differences in phosphorylation between the phenotypes. This is consistent with resistance to Varroa mite infestation being a complex and multi-faceted process.

Specific consideration of these differentially phosphorylated peptides from the perspective of gene ontology and pathway overrepresentation analysis revealed a number of points of biological difference between uninfested bees of the resistant and susceptible phenotypes (Table 2 and Supplementary Table 2), between infested and uninfested bees of the susceptible phenotype (Table 3 and Supplementary Table 3), and between infested and uninfested bees of the resistant phenotype (Table 4 and Supplementary Table 4). When comparing uninfested bees from the two phenotypes, there were no clear differences in pathways and processes associated with immune function (Table 2 and Supplementary Table 2). An interesting exception is that within the G4 pupae, there was a trend toward the down-regulation of innate immunity (*P*-value < 0.1) in response to Varroa mite infestation (Table 3). Down-regulation of innate immune processes in response to Varroa mite infestation was not observed in the resistant phenotype (Table 4).

**Table 2:** **Gene ontology analysis of uninfested resistant and susceptible dark-eyed pupae (S88-/G4-)**.

**Category**	**Name**	**ID**	**1**	**2**	**3**	**4**	**5**
Biological process	Cell cycle arrest	GO:0007050	5	5	0.040	0	1
	Response to peptide hormone stimulus	GO:0043434	4	4	0.078	0	1
	ATP biosynthetic process	GO:0006754	4	0	1	4	0.03
	Positive regulation of neuron apoptosis	GO:0043525	4	0	1	4	0.03
	Cytoskeleton organization	GO:0007010	6	1	0.99	5	0.05
Cellular component	Cell surface	GO:0009986	7	6	0.082	1	0.98
	Golgi apparatus	GO:0005794	7	1	0.99	6	0.02

*Based on levels of differential expression or phosphorylation, InnateDB (Lynn et al., 2008) can predict upregulated or downregulated pathways that are consistent with the experimental data. Pathways are assigned a P-value based on the number of proteins present for a particular pathway. The numbered columns are as follows: 1, total genes uploaded for that pathway; 2, number of genes up-phosphorylated; 3, P-value for up-phosphorylation; 4, number of genes down-phosphorylated; 5, P-value for down-phosphorylation*.

**Table 3:** **Gene ontology analysis of susceptible dark-eyed pupae (G4+/G4−)**.

**Category**	**Name**	**ID**	**1**	**2**	**3**	**4**	**5**
Biological process	Transport	GO:0006810	4	0	1	4	0.081
	Innate immune response	GO:0045087	27	9	0.945	18	0.098
	Cell cycle	GO:0007049	9	1	0.99	8	0.03
	DNA repair	GO:0006281	5	0	1	5	0.04
	Mitotic cell cycle	GO:0000278	5	0	1	5	0.04
	Glycolysis	GO:0006096	7	6	0.031	1	0.99
	Phosphatidylinositol-mediated signaling	GO:0048015	4	4	0.039	0	1
	Multicellular organismal development	GO:0007275	6	5	0.064	1	0.992
Cellular component	Nucleoplasm	GO:0005654	22	7	0.95	15	0.106
	Plasma membrane	GO:0005886	30	17	0.099	13	0.94
	Golgi apparatus	GO:0005794	8	1	0.99	7	0.05
	Integral to membrane	GO:0016021	8	6	0.081	2	0.98
	Basolateral plasma membrane	GO:0016323	4	4	0.038	0	1
Molecular function	ATPase activity	GO:0016887	4	0	1	4	0.081
	RNA binding	GO:0003723	4	0	1	4	0.081
	RNA pol. II transcription factor activity	GO:0003705	4	4	0.038	0	1

For details, see the caption and footnote of Table 2.

**Table 4:** **Gene ontology analysis of resistant dark-eyed pupae (S88+/S88−)**.

**Category**	**Name**	**ID**	**1**	**2**	**3**	**4**	**5**
Biological process	RNA metabolic process	GO:0016070	5	1	0.99	4	0.067
	mRNA metabolic process	GO:0016071	5	1	0.99	4	0.067
	Nerve growth factor receptor signaling	GO:0048011	7	7	0.028	0	1
	Positive regulation of apoptotic process	GO:0043065	5	5	0.082	0	1
	Peptidyl-serine phosphorylatio	GO:0018105	7	2	0.99	5	0.069
Molecular function	Phosphor-transferase activity	GO:0016772	15	12	0.087	3	0.978
	Protein kinase binding	GO:0019901	4	0	1	4	0.018
	RNA binding	GO:0003723	5	1	0.99	4	0.067
	DNA binding	GO:0003677	7	2	0.99	5	0.069
	Kinase activity	GO:0016301	14	6	0.97	8	0.094

For details, see the caption and footnote of Table 2.

### 3.7. Detection of secondary viral infections

**Figure 5:**
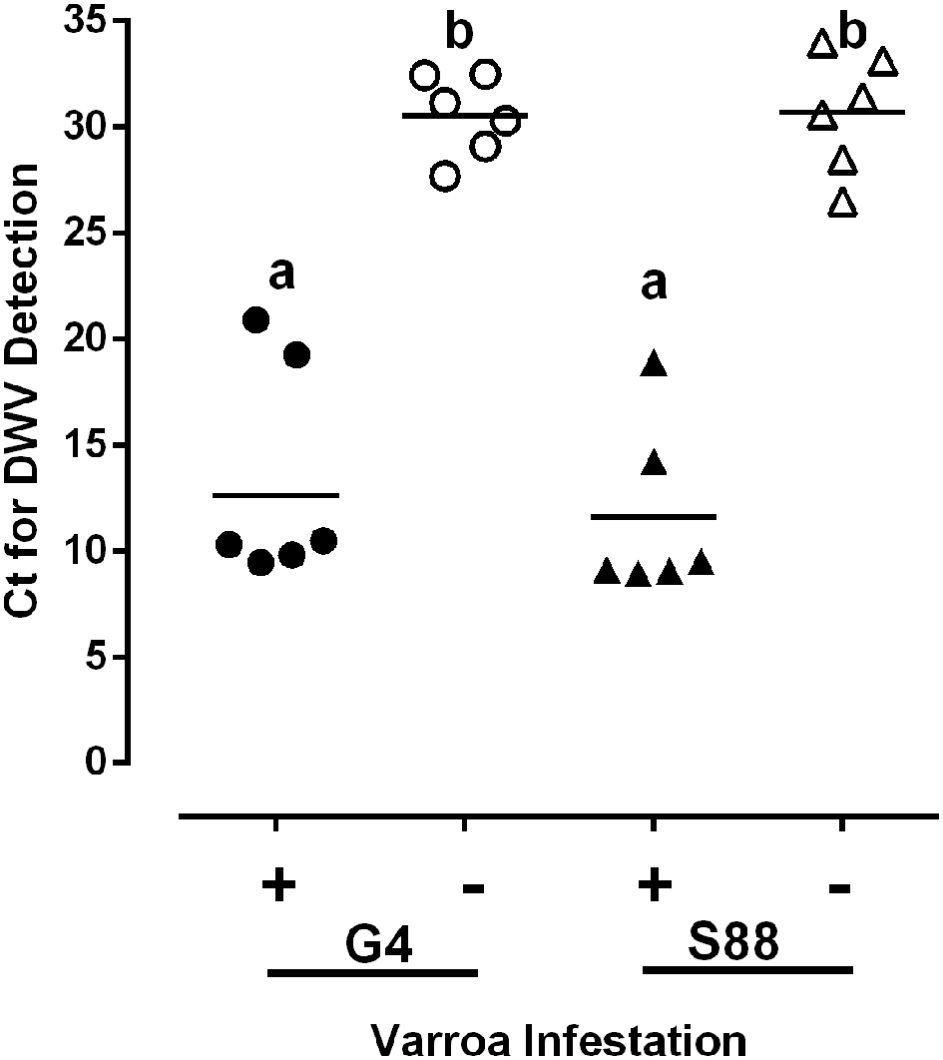
**Virus presence in honeybee populations**. The level of deformed wing virus (DWV) present in dark-eyed pupae was compared in the presence (+) or absence (−) of a detectable Varroa mite infestation. DWV was detected using qRT-PCR and the level of viral infection was measured as the threshold cycle (Ct) for viral RNA amplification. Ct values are inversely proportional to the abundance of viral RNA. Data presented are values for individual pupae (*n* = 6/group). Significant differences (*P*-value < 0.05) among treatment groups are denoted by different letters above each column.

For bees of both phenotypes, at the dark-eyed pupae stage of development and in the absence of Varroa mites, there was a shared presence of detectable, but low levels of DWV (Figure 5). However, in the presence of Varroa mites there was an approximately 10,000-fold increase in DWV RNA relative to the Varroa mite-free pupae (Figure 5). There was also no detectable IAPV and KBV RNA in pupae of both phenotypes, regardless of the presence or absence of mite infestation (data not shown). These observations support the hypothesis that Varroa mites serve as a vector for virus transmission and that both phenotypes experience equal levels of viral infection following mite infestation. This observation supports the conclusion that kinotypic differences between pupae from the two phenotypes reflect differences in host responses to the Varroa mite and not viral infection.

The presence of immunosuppression was suggested by kinome data analysis of susceptible bees at the dark-eyed pupae stage of development. If this immunosuppression persists throughout the life of a bee, then the ability of bees to counter further infection by secondary pathogens may be compromised. Consistent with this hypothesis, screening for two additional viral bee pathogens, IAPV and KBV, confirmed higher rates of infection in the susceptible adult bees in the face of Varroa mite infestation (Table 5).

**Table 5:** **Percentage of resistant and susceptible adult bees with detectable virus**.

**Virus**	**G4 (%)**	**S88 (%)**
DWV	100	100
IAPV	60	0

Bees (n = 20/group) were sampled in September 2010 (see Figure 1A). Viruses were detected using 30 cycles of amplification in qRT-PCR, and amplified products were visualized by agarose gel electrophoresis. Specific primer pairs were used to detect deformed wing virus (DWV), Israeli acute paralysis virus (IAPV), and Kashmir bee virus (KBV).

## 4. Discussion

There is a clear and emerging priority for the ability to define global host responses at the level of phosphorylation-mediated signal transduction. As technologies advance, there is greater opportunity to apply these approaches to a broader range of species as well as samples of increasing biological complexity. Kinome analysis is often performed on cellular samples of low complexity, such as cultured cells, or purified primary cell populations, such as monocytes. Recently, there have been demonstrations of kinome analysis of samples of greater biological complexity, such as organ samples (Arsenault et al., 2013b) and intestinal tissue (Määttänen et al., 2013). The current report, to the best of our knowledge, represents the first development of an insect-specific peptide kinome array as well as the first application of kinome analysis at the whole-organism level. The incentive to push the technology in this direction was to develop a research tool of value in the understanding of colony collapse disorder of bees. Specifically, we sought to apply the bee-specific array to populations with differing resistance to Varroa mite infestation, in the presence and absence of this critical pathogen, to provide insight into mechanisms of disease resistance as well as biomarkers for strategic bee breeding programs.

The kinome data emerging from analysis of distinct phenotypes (susceptible and resistant) at three developmental stages (pink-eyed pupae, dark-eyed pupae, and adults) provided clear evidence of a phenotype-associated kinotype. As might be anticipated, each stage of development was also associated with a different global pattern of signal transduction activity. Within these development-specific patterns of clustering, there was clear evidence for distinct sub-profiles corresponding to each of the Varroa mite susceptibility phenotypes. This suggests the potential to translate the arrays into a tool that could be utilized to inform commercial aspects of bee production, such as sales and breeding. Phosphosignatures that reflect important phenotypes, such as disease resistance or production value, could be incorporated into a second generation honeybee-specific array.

In the absence of Varroa mite infestation, there were clear and consistent differences in the signaling profiles of the susceptible and resistant bees. The magnitude of these differences suggests that resistance is a complex, multifactorial process. Interestingly, for the uninfested bees there were no obvious differences between the two phenotypes that relate to pathways or processes immediately associated with immunity. This is consistent with a previous investigation of the biological basis of Varroa mite susceptibility phenotypes through gene expression approaches, which suggested that differences in behavior, rather than immune function, underlie Varroa resistance (Navajas et al., 2008). The most well-defined traits associated with Varroa resistance are hygienic behavior and grooming behavior that function to maintain lower Varroa populations (Harbo and Harris, 2009; Tsuruda et al., 2012). The S88 phenotype also showed better grooming behavior (unpublished observations). However, in our breeding efforts, it is difficult to stabilize Varroa resistant phenotypes, and the progeny of selected colony phenotypes are highly variable. Colony phenotypes can also change over time within the same colony. The survival of a resistant phenotype may be due to combinations of grooming and hygienic behavior as well as undefined mechanisms that restrict the propagation of viral pathogens. This combination of traits may be critical for bee survival in the presence of a persistent Varroa infestation. Elucidation of the mechanisms involved in this resistance to colony collapse may be critical for breeding bees able to tolerate low levels of persistent Varroa parasitism while maintaining colony health.

The responses of the two bee phenotypes to Varroa mite infestation in the current study were also investigated using pathway over-representation and gene ontology analysis. For the resistant bees, a small number of pathways were found to be activated in response to Varroa infestation. Specifically, there was robust activation of MAPK signaling, which may represent the most effective host response through induction of stress response pathways. Activation of MAPK signaling has been linked to successful management of pathogenic challenge in a number of species, including insects (Arthur and Ley, 2013). In contrast, within the susceptible bees, there were more far-reaching consequences to Varroa mite challenge, including evidence for a down-regulation of innate immune responses.

There are conflicting opinions in the literature regarding the significance of host immunity, and the potential ability of Varroa mites to compromise host immunity. For example, some investigations have reported that Varroa mites, or virus associated with mites, compromise honeybee immunity (Gregory et al., 2005) and promote amplification of bee viruses (Yang and Cox-Foster, 2005). From a more global perspective, a number of ectoparasites immunosuppress their vertebrate hosts and increase susceptibility to infectious disease (Yang and Cox-Foster, 2005). Varroa mites may contribute to colony collapse by suppressing bee immunity and promoting secondary viral infections (Yang and Cox-Foster, 2005; Evans and Schwarz, 2011). Given the conserved transmission route associated with many bee parasites, co-infection of individual bees and colonies by multiple viral pathogens is a common occurrence that can have direct and indirect interactions that may be additive, synergistic or neutral in consequences to the host (Evans and Schwarz, 2011). Varroa mites are associated with a number of honeybee RNA viruses. In this capacity, the mites are known to contribute to colony failure both by acting as a reservoir and incubator for the viruses as well as facilitating their spread among bees (Nazzi et al., 2012). Our work adds another layer to this synergy by suggesting that infestation by the mite renders the bee host more susceptible to viral infection by compromising the innate immune system.

Our kinome data strongly indicate that differences in immune capabilities are likely not involved in Varroa susceptibility; rather, this phenotype may reflect primarily behavioral differences. Following Varroa mite infestation, however, the immunosuppression observed in the susceptible bees may influence their ability to counter further infestation by mites as well as secondary viral pathogens. This hypothesis is supported by greater diversity of secondary viral infections in the susceptible bees following Varroa mite infestation. This could occur at the level of the individual bees as well as the entire colony. The ultimate collapse of these colonies may represent the collective toll of these combined infections, as well as other potential stressors. This suggests that bees are not susceptible to Varroa mite infestation because they are immunocompromised; rather, they are immunocompromised because they are infested with Varroa mites. This understanding, in concert with the use of the arrays to identify appropriate biomarkers, may enable strategic breeding and management efforts to deal with the problem of Varroa parasitism and honeybee colony loss worldwide.

This initial kinome-wide analysis of honeybees has generated a number of important questions that motivate further experimental investigation. For example, more targeted investigation of the host-pathogen interaction between honeybees and Varroa mites may confirm the hypothesis that the vulnerability of the susceptible bees reflects consequences of Varroa mite infestation, as well as evidence of the molecular mechanisms involved. Unknown factors may be acting at the cellular level in Varroa resistant bees identified by natural selection (survival colonies), which may or may not be present in bees showing behavioral characteristics for expression of Varroa resistance. These factors may protect against the fatal effects associated with viruses (DWV, IAPV, KBV) vectored by Varroa, or may reduce the ability of Varroa to cause deficiencies in innate immune or stress responses. Experiments are in progress using honeybee kinome analyses to investigate these possibilities in individual bees from inbred colony lines showing varying degrees of resistance and susceptibility to Varroa. Additionally, the ability of the proposed phosphorylation-associated biomarkers of Varroa mite susceptibility should be evaluated in large-scale investigations of honeybees representing a spectrum of susceptibilities. The ability of these markers to effectively discriminate and predict this important phenotype within the context of naturally occurring variance will be important for determining the value of these markers. Ultimately, a methodology for using specific, targeted subsets of the peptide array probes (just 5–10 of them) to identify Varroa resistant and susceptible phenotypes needs to be developed.

## Author contributions

Albert J. Robertson designed the breeding program, helped plan the kinome array experiments, participated in data analysis, supervised the research, and wrote parts of the manuscript. Brett Trost helped design the honeybee-specific kinome arrays, participated in data analysis, and wrote parts of the manuscript. Erin Scruten performed the kinome array experiments. Thomas Robertson and Mohammad Mostajeran performed bee selections and helped define the resistant and susceptible phenotypes. Wayne Connor performed the virus quantification experiments. Anthony Kusalik and Philip Griebel participated in data analysis and supervised the research. Scott Napper helped design the honeybee-specific arrays, participated in data analysis, wrote parts of the manuscript, and supervised the research. All authors participated in revising the manuscript, with particular contributions by Albert J. Robertson, Brett Trost, Anthony Kusalik, and Scott Napper.

## Funding

This work was funded in part by grants from Saskatchewan Agriculture (Agriculture Development Fund) and the Agriculture Council of Saskatchewan to Albert J. Robertson and by Meadow Ridge Enterprises Ltd. Philip Griebel holds a Tier I Canada Research Chair funded by the Canadian Institutes of Health Research. Brett Trost and Anthony Kusalik received funding from the Natural Sciences and Engineering Research Council of Canada (NSERC).

### Conflict of interest statement

The authors declare that the research was conducted in the absence of any commercial or financial relationships that could be construed as a potential conflict of interest.
