# Supplementary material for: Identification of developmentally-specific kinotypes and mechanisms of Varroa mite resistance through whole-organism, kinome analysis of honeybee
Source: Front Genet. 2014 May 21;5:139. doi: 10.3389/fgene.2014.00139 (PMC4033134; doi:10.3389/fgene.2014.00139)
Supplement: Supplementary file 1 [file DataSheet1.PDF]

Frontiers in Genetics

Research Article
20147 April 2014

Supplementary Material: Identification of developmentally-specific kinotypes and mechanisms of Varroa mite resistance through whole-organism, kinome analysis of honeybee

Albert J Robertson [1], Brett Trost [2], Erin Scruten [3], Thomas Robertson [1], Mohammad Mostajeran [1], Wayne Connor [3], Anthony Kusalik [2], Philip Griebel [3,4] and Scott Napper [3,5,*]

[1] *Meadow Ridge Enterprises Ltd, Box 1, Group site 602, RR6, Saskatoon, Saskatchewan, Canada*
[2] *Department of Computer Science, University of Saskatchewan, Saskatoon, Saskatchewan, Canada*
[3] *Vaccine and Infectious Disease Organization, University of Saskatchewan, Saskatoon, Saskatchewan, Canada*
[4] *School of Public Health, University of Saskatchewan, Saskatoon, Saskatchewan, Canada*
[5] *Department of Biochemistry, University of Saskatchewan, Saskatoon, Saskatchewan, Canada*

Correspondence*:
Scott Napper
Vaccine and Infectious Disease Organization, University of Saskatchewan, Saskatoon, Saskatchewan, S7N 5E3, Canada, scott.napper@usask.ca

1 SUPPLEMENTARY DATA

Supplementary File 1 (honeybee_array.gal) - GenePix Array List (GAL) file containing the exact layout and content of the honeybee array used in this study.

2 SUPPLEMENTARY TABLES AND FIGURES

Supplementary Table 1. Using sequence homology to identify honeybee phosphorylation sites. The first column indicates the number of sequence differences between a known phosphorylation site from the PhosphoSitePlus or Phospho.ELM database, and its best match in the honeybee proteome. The second column represents, for all sites in these databases, the percentage that had that number of sequence differences. The third column represents the percentage of peptides actually chosen for inclusion on the array having a given number of sequence differences.

Sequence Differences	All query peptides	Peptides on the array
0	0.6%	12.7%
1	0.8%	21.7%
2	1.1%	16.4%
3	1.2%	19.7%
4	1.4%	12.4%
5	1.6%	7.7%
6	1.6%	6.0%
7	1.3%	2.7%
8+ or no match	90.4%	0.7%

Supplementary Table 2. Pathway analysis of peptides differentially phosphorylated between resistant and susceptible uninfested bees (S88-/G4-). The columns are as follows: 1, total number of peptides; 2, number of upregulated peptides; 3, p-value for upregulation; 4, number of downregulated peptides; 5, p-value for downregulation.

Pathway Name	1	2	3	4	5
Hypoxia and p53 in the cardiovascular system	4	0	1	4	0.024
HIF-1-alpha transcription factor network	5	0	1	5	0.0089
Vegf hypoxia and angiogenesis	5	0	1	5	0.0089
Epithelial cell signaling in Helicobacter pylori infection	6	1	0.99	5	0.037
Hypoxia-inducible factor in the cardivascular system	3	0	1	3	0.062
P38 mapk signaling pathway	4	0	1	4	0.024
MAPK signaling pathway	21	8	0.98	12	0.062
Links between pyk2 and map kinases	9	2	0.99	6	0.090
Chemokine signaling pathway	12	3	0.99	8	0.047
IL2-mediated signaling events	4	0	1	4	0.024
CXCR4-mediated signaling events	6	0	1	6	0.0032
RAC1 signaling pathway	8	0	1	8	0.0004
Focal adhesion	13	2	0.99	10	0.0047
Signaling events mediated by Hepatocyte Growth Factor Receptor	9	1	0.99	8	0.0025
Endocytosis	7	7	0.013	0	1
CDC42 signaling events	11	2	0.99	9	0.0038
AndrogenReceptor	5	0	1	5	0.0089
Endothelins	5	0	1	5	0.0089
Integrin-linked kinase signaling	5	0	1	5	0.0089
ErbB2/ErbB3 signaling events	7	1	0.99	6	0.016
Class I PI3K signaling events mediated by Akt	4	0	1	4	0.024
Downstream signaling in nave CD8+ T cells	4	0	1	4	0.024
S1P2 pathway	4	0	1	4	0.024
p75(NTR)-mediated signaling	4	0	1	4	0.024
Wnt signaling pathway	6	1	0.99	5	0.037
Signaling events mediated by VEGFR1 and VEGFR2	8	2	0.98	6	0.044
VEGF signaling pathway	10	2	0.99	7	0.047
AP-1 transcription factor network	3	0	1	3	0.062
Aurora A signaling	3	0	1	3	0.062
CXCR3-mediated signaling events	3	0	1	3	0.062
Carbohydrate digestion and absorption	3	0	1	3	0.062
Cell to cell adhesion signaling	3	0	1	3	0.062
DSCAM interactions	3	0	1	3	0.062
E-cadherin signaling in the nascent adherens junction	3	0	1	3	0.062
IL6-mediated signaling events	3	0	1	3	0.062
Integrin signaling pathway	3	0	1	3	0.062
N-cadherin signaling events	3	0	1	3	0.062
Nephrin/Neph1 signaling in the kidney podocyte	3	0	1	3	0.062
Sema4D induced cell migration and growth-cone collapse	3	0	1	3	0.062
TNFalpha	19	7	0.98	11	0.068
Glucocorticoid receptor regulatory network	5	1	0.98	4	0.083
LPA receptor mediated events	5	1	0.98	4	0.083

Supplementary Table 2 (continued).

Pathway Name	1	2	3	4	5
LPA receptor mediated events	5	1	0.98	4	0.083
Leukocyte transendothelial migration	5	1	0.98	4	0.083
Rac1 cell motility signaling pathway	5	1	0.98	4	0.083
Ras signaling pathway	5	1	0.98	4	0.083
Reelin signaling pathway	5	1	0.98	4	0.083
Signaling events regulated by Ret tyrosine kinase	5	1	0.98	4	0.083
Wnt	11	3	0.99	7	0.088
Agrin in postsynaptic differentiation	7	1	0.99	5	0.090
Bcr signaling pathway	7	1	0.99	5	0.090
Trk receptor signaling mediated by the MAPK pathway	7	2	0.97	5	0.090
Alpha6Beta4Integrin	9	1	0.99	6	0.090
ErbB1 downstream signaling	9	3	0.96	6	0.090

Supplementary Table 3. Pathway analysis of peptides differentially phosphorylated between infested and uninfested susceptible bees (G4+/G4-). Columns are as in Supplementary Table 2.

Pathway Name	1	2	3	4	5
CDC42 signaling events	7	6	0.02	1	0.99
Signaling events mediated by Hepatocyte Growth Factor Receptor	7	6	0.02	1	0.99
Integrins in angiogenesis	4	4	0.03	0	1
Colorectal cancer	8	6	0.07	2	0.99
Pathways in cancer	18	11	0.08	7	0.97
Ctcf: first multivalent nuclear factor	3	3	0.08	0	1
Downstream signaling in nave CD8+ T cells	3	3	0.08	0	1
Endothelins	3	3	0.08	0	1
Integrin-linked kinase signaling	3	3	0.08	0	1
P38 mapk signaling pathway	3	3	0.08	0	1
Pancreatic secretion	3	3	0.08	0	1
Phagosome	3	3	0.08	0	1
Regulation of Androgen receptor activity	3	3	0.08	0	1
Regulation of retinoblastoma protein	3	3	0.08	0	1
RhoA signaling pathway	3	3	0.08	0	1
Signaling events mediated by HDAC Class III	3	3	0.08	0	1
Validated nuclear estrogen receptor alpha network	3	3	0.08	0	1
EGFR1	31	17	0.09	14	0.95
Glycolysis and Gluconeogenesis	5	4	0.11	1	0.99
RAC1 signaling pathway	5	4	0.11	1	0.99
Signaling events mediated by VEGFR1 and VEGFR2	5	4	0.11	1	0.99
Oocyte meiosis	6	0	1	6	0.03
FOXM1 transcription factor network	4	0	1	4	0.09
IL-1 signaling pathway (through p38 cascade)	4	0	1	4	0.09
IL-7	4	0	1	4	0.09
IL-9	4	0	1	4	0.09
Interleukin-1 signaling	4	0	1	4	0.09
T cell receptor signaling pathway	10	2	0.98	8	0.09
Signal transduction by L1	7	1	0.98	6	0.09

Supplementary Table 4. Pathway analysis of peptides differentially phosphorylated between infested and uninfested resistant bees (S88+/S88-). Columns are as in Supplementary Table 2.

Pathway Name	1	2	3	4	5
Pathways in cancer	10	9	0.036735	1	0.996344
MAPK signaling pathway	15	12	0.068044	3	0.98515
